# The dual-hormone hypothesis and first-time fathers’ relationship satisfaction at 3 months postpartum

**DOI:** 10.3389/fpsyg.2025.1447640

**Published:** 2025-05-22

**Authors:** Rylei L. Donovan, Randy Corpuz

**Affiliations:** ^1^Department of Psychology, University of Massachusetts Boston, Boston, MA, United States; ^2^Department of Psychology, Pepperdine University, Malibu, CA, United States

**Keywords:** fathers, testosterone, cortisol, dual hormone, relationships

## Abstract

Human males face tradeoffs in how they invest resources toward mating and parenting. Research on male’s transition to fatherhood has revealed shifts in hormones tied to these tradeoffs. While work has focused on the influence of hormones on parenting during this stage, less is known about how these hormones influence mating (i.e., relationship functioning with partner) in the postnatal period. A father’s relationship satisfaction is expected to be related to endocrine activity across the transition to parenthood. We predicted that first-time fathers with high testosterone (T) would report lower relationship satisfaction. We expected this effect to be amplified (moderation) for those males with lower cortisol (CORT) levels (i.e., dual hormone hypothesis). At 3 months postpartum we measured salivary T and CORT (*n* = 220) and recorded relationship satisfaction using the Investment Model Scale (IMS). We found that fathers with high T and low CORT had the highest relationship satisfaction. While the effect was small, these findings ran counter to our predictions. We speculate that higher T and lower CORT males may report increased satisfaction as they support, retain, and secure additional opportunities from a mate who recently demonstrated her ability (and willingness) to produce offspring. Discussion focuses on numerous limitations of the study, small effect size, and the need for replication with less homogenous samples.

## Overview

Psychobiological research has progressed from studying the functional role of single hormones to a more nuanced understanding of how hormones interact to facilitate behavior. Recent work using the dual hormone hypothesis (DHH) finds that testosterone (T) and cortisol (CORT) interact to regulate behaviors in specific domains (Mehta and Josephs, 2010). According to the DHH, positive associations between T and behaviors conventionally associated with dominance, competition, and status-seeking increase when CORT levels are low (Mehta and Prasad, 2015; see Dekkers et al., 2019 for meta-analysis). While research on the association between individual hormones and romantic relationships has grown in recent years (see Edelstein, 2022 for review), predictions using the DHH remain untested in the domain of romantic relationships. Extant DHH work has also yet to fully leverage known windows of neuroendocrine fluctuations in baseline levels of CORT and/or T—e.g., first-time fatherhood (Gettler et al., 2011; Saxbe et al., 2017a; Saxbe et al., 2017b; Kuo et al., 2018)—and, instead, focus (mostly) on hormone reactivity in response to laboratory tasks/stimuli (Dekkers et al., 2019). In this study, we focus on the transition to first time fatherhood and the role of T, CORT, and the DHH in a new father’s relationship satisfaction with his partner.

## Literature review

### T and relationships

T is the major output of the hypothalamic–pituitary-gonadal (HPG) axis and fluctuates in response to developmental milestones such as forming new romantic relationships or the transition to parenthood (Grebe et al., 2019). The role of T in forming and maintaining relationships is a common focus of psychobiological work (Saxbe et al., 2017a; Saxbe et al., 2017b; Cárdenas et al., 2023; see Edelstein, 2022). Research has also focused on T as facilitating male dominance and aggression as well as being antagonistic to forming/maintaining long term pair bonds (Gray et al., 2020). For example, T is associated with more lifetime sexual partners and decreased relationship satisfaction (Dhillon et al., 2020; van der Meij et al., 2019; Tackett et al., 2014; Welker et al., 2014). Edelstein et al. (2014) measured relationship satisfaction and commitment in heterosexual couples and salivary T. Findings suggested that a woman’s relationship satisfaction is negatively associated with their partner’s T. The idea that T is antagonistic to nurturing behavior is commonly presented as an explanation for why higher T has been correlated with lower relationship satisfaction. Dhillon et al. (2020) examined associations between T, perceived partner accommodation, and conversation satisfaction during a relationship stressor task. The findings of this study found that high T was negatively related to perceived partner accommodation during the task. Overall, evidence suggests that high T is negatively related to relationship functioning.

### CORT and relationships

CORT is produced by the hypothalamic–pituitary–adrenal (HPA) axis and is often examined in association with depression, anxiety, aggression, and stress (Gordis et al., 2006; Galbally et al., 2019). More recently, CORT has also been found to be related to relationship support and nurturing behaviors. For example, paternal CORT is significantly lower in fathers than non-father male controls (Berg and Wynne-Edwards, 2001). Lower CORT is also related to higher quality caregiving for fathers (Beijers et al., 2022). Other literature suggests that higher paternal CORT on the day of their infant’s birth, the day following, and after first holding their infant is correlated with more paternal care (Kuo et al., 2018). Braren et al. (2020) found that, during pregnancy, low paternal CORT acts as a buffer for maternal CORT in mothers that report high stress. Taken together, high CORT is negatively correlated with relationship functioning. While CORT—relative to T—is not as commonly studied for its relationship with supportive and nurturing behaviors, research on CORT moderating T in close relationships is (to our knowledge) wholly absent from the literature to date.

### T × CORT interaction

Aside from CORT, the HPA axis is also regulated by androgens—most notably T (Bingham et al., 2011; Chen et al., 2014). For example, androgens have both activational and inhibitory actions on the HPA axis (Zuloaga et al., 2024). The integrated and reciprocal interactions between the HPA and HPG axes has been documented through endocrine manipulations in animal research (Viau, 2002). For example, increases in CORT have inhibitory effects on the release of sex steroids specific to mating and reproduction (Tilbrook et al., 2000). Conversely, male T inhibits the HPA axis’ response to relational stressors (Viau and Meaney, 1996). Altogether, these interactions suggest the possibility of a T × CORT interaction being related to romantic relationships where the HPA and HPG axes have been implicated (separately) in humans.

The DHH suggests that the influence of T on behavior is amplified when levels of CORT are low (Mehta and Josephs, 2010). The idea that CORT modulates the androgenicity of T has generated novel findings of varying effect sizes (see Dekkers et al., 2019). However, discoveries are constrained to categories of behavior (e.g., dominance) putatively antagonistic to maintaining close relationships in socially monogamous, paternally investing species like humans (Donovan et al., 2023) and have yet to generate systematic research in domains related to close relationships. As noted above, elevated levels of T are generally associated with poor relationship satisfaction. It is an open question as to whether T’s negative association with relationship satisfaction may be more pronounced among human males who also have lower levels of CORT.

### Hypotheses

In integrating literature on T, CORT, and relationship functioning with extant DHH research, our primary prediction is that relationship satisfaction will be lowest in individuals with high T and low CORT (i.e., CORT will moderate the relationship between T and relationship satisfaction). Aligned with the DHH literature, we also test for main effects of T and CORT (individually) on relationship satisfaction. We predict that males with high T will report lower satisfaction in their romantic relationships and that males with high CORT will also have lower satisfaction in their romantic relationships.

## Methods

### Overview and study design

The secondary data used in this study is from a previously completed longitudinal study on paternal postpartum health outcomes for first-time fathers (see Corpuz et al., 2021). All data used for the current report was collected approximately 3 months following childbirth (*M* = 96.07 days, *SD* = 16.48 days). All materials and procedures were reviewed and approved by the University’s Institutional Review Board (IRB). Participants were provided information on the risks and benefits of participating in this research, signed consent forms prior to data collection, and were compensated for their contributions. The data for this study was collected between 2013 and 2015 and all saliva assays (T and CORT) were conducted between 2014 and 2015.

### Participants

First-time fathers (*n =* 220) completed self-report measures and submitted saliva samples. Fathers were recruited from multiple sources: hospital birthing or community lactation classes (62.7%), midwife referrals (15.7%), social media ads (13.6%), or community Baby Basics class (2.2%). The remaining 6% of the sample did not report a recruitment source. All participants were residing in Southern California (U.S.A.) at the time of data collection.

As noted in Corpuz et al. (2021), the average age of fathers in this study was *M* = 32.9, *SD* = 5.4, 84.1% of this sample was married to their child’s mother (all but one couple reported cohabitating at time of study) and 77.4% of these fathers held at least a college degree. The median income of this sample was $50,000 to $75,000. Fathers self-reported their race/ethnicity as White (70.6%), Latino/Hispanic (12%), Asian American (5.2%), Black/African American (1.7%), Native American (1.3%), multiracial (2.6%), and other (3.9%).

### Materials and procedure

Participants completed self-report questionnaires during pre-planned home visits. Following the completion of self-report measures, home visitors trained fathers on how to expectorate saliva through a simulated collection procedure using the exact materials they would use on the day of sampling. Parents were provided with pre-labeled saliva kits (sterile cotton swabs, polypropylene tubes, written instructions, and Ziploc bags) and a video demonstrating the process in detail.

#### Saliva collection

Fathers were instructed to expectorate saliva “within 30 min of waking up” during their next day off from work where applicable (i.e., a weekend day for most parents; see Corpuz et al., 2021). The specific day of sample selection (and subsequent sample retrieval) was agreed upon between the home visitor and the participant. Mean sampling times across participants was 6:47 am (SD = 1:09).

Fathers were told to abstain from alcohol (12 h prior), all food (1 h prior), and any beverages containing sugar, acid, or caffeine (5 min prior) leading up to their morning sample as per Granger et al. (2012). During sampling, fathers placed a sterilized absorbent cotton swab underneath their tongue for a minimum of 120 s. They then directed the swab into a polypropylene tube (using their tongue) and placed the tube into a freezer safe bag and into the freezer until samples were retrieved by a home visitor. Home visitors retrieved saliva samples from parents within 7 days of each visit^1^. All samples were retrieved from participants, inventoried, and frozen at−50°C for up to 90 days and were then shipped on dry ice.

#### Saliva assays

Samples were assayed in duplicate at the Institute for Interdisciplinary Salivary Bioscience Research (IISBR; Arizona State University) using a highly sensitive competitive enzyme immunoassay (EIA) without modifications to the recommended protocols from Salimetrics (Carlsbad, CA).

#### Testosterone (T)

The test volume for T assay was 25 μL, and range of sensitivity was from 1.0 to 600 pg./mL. On average the inter- and intra-assay coefficients of variation were less than 15 and 10%, respectively. Reagents were stored at 2–8 degrees (C); reagents and samples were completed without interruption across a 96-well microtiter plate coated with polyclonal anti-T antibodies. The full assay protocol can be downloaded directly from the manufacturer: https://salimetrics.com/wp-content/uploads/2018/03/testosterone-saliva-elisa-kit.pdf.

#### Cortisol (CORT)

For CORT, the assay range of sensitivity was 0.004–3.0 μg/dL. The detection limit was 0.018 μg /dl (after accounting for extraction dilution). On average the inter- and intra-assay coefficients of variation were less than 10 and 5%, respectively. Reagents were stored at 2–8 degrees (C); reagents and samples were completed without interruption across a 96-well microtiter plate coated with monoclonal anti-CORT antibodies. The full assay protocol can be downloaded directly from the manufacturer: https://salimetrics.com/wp-content/uploads/2018/03/salivary-CORT-elisa-kit.pdf.

#### Missing data: T and CORT

Eleven fathers were missing data for both CORT and T (six fathers provided insufficient quantity; four fathers had a concentration below lower limit of sensitivity; one father dropped out of the study prior to saliva retrieval). These cases are retained in analyses following maximum likelihood estimation to address missingness for CORT and T. There were two fathers with outlying values (> 3SDs) for CORT and one father with an outlying value for T. These three cells were replaced as missing and retained in analyses following maximum likelihood estimation to address missingness for these variables (see Corpuz et al., 2021).

#### Relationship satisfaction

To measure relationship satisfaction participants completed the satisfaction subscale of the Investment Model Scale (IMS; Rusbult et al., 1998). This relationship satisfaction subscale is a widely used self-report questionnaire with high internal consistency and acceptable validity in the literature (Edelstein et al., 2014; Saxbe et al., 2017a; Saxbe et al., 2017b). The subscale is composed of 10 items scored on a Likert scale. The first five items have scores from 0: “Do not Agree At All” to 4: “Agree Completely” with items such as “My partner fulfills my needs for companionship (doing things together, enjoying each other’s company, etc.)” and “My partner fulfills my needs for security (feeling trusting, comfortable in a stable relationship, etc.).” The last five items have scores from 0: “Do Not Agree At All” to 8: “Agree Completely” with items such as “My relationship is much better than others’ relationships” and “My relationship is close to ideal.” In the current sample of fathers, the scale was highly reliable (Cronbach’s^2^ *α* = 0.93).

## Results

Primary analyses for this report were executed using a structural equation modeling (SEM) framework which includes a robust maximum likelihood estimation (MLE) missing data module in AMOS v.27 (IBM Chicago; Arbuckle, 2019).

### Covariates

#### Demographic covariates

No differences were observed in study variables due to marital status (*p*s>0.79), household income (*p*s>0.61), or self-reported ethnicity (*p*s>0.68).

#### Endocrine covariates

A series of bivariate correlations were tested to evaluate covariates^3^ for inclusion in models: BMI, father’s age, and time of morning saliva sample was collected. Fathers’ BMI was not associated with their morning T (*r* = −0.01, *p* = 0.93) nor morning CORT (*r* = −0.02, *p* = 0.74). While paternal age was not related to T (*r* = −0.07, *p* = 0.32), it was significantly correlated with morning CORT (*r* = 0.20, *p* = 0.004) and, as a result, age was included as a covariate in subsequent models that included morning CORT. Exact time of morning sample was not related to father’s morning T (*r* = 0.04, *p* = 0.54) but was correlated with paternal morning CORT (*r* = −0.15, *p* = 0.04). Subsequent CORT models include time of morning sample as a covariate.

There were 27 fathers that self-reported smoking tobacco which can influence salivary assay values (see Granger et al., 2012). However, smoking status was not related to T (*p* = 0.18) nor CORT (*p* = 0.63) in this sample of fathers. Three fathers reported taking medications with documented effects on either CORT or T production (e.g., aromatase inhibitor). We elected for a conservative approach to handling these medications in our analyses; values for T and CORT for all three fathers were removed and replaced as missing^4^.

#### Missing data

Overall, missingness for the variables tested in this study were moderate (0–6.1%) (Little and Rubin, 2019). To adjust for biases due to missing data, we fitted all models using the maximum likelihood estimation (MLE) missing data module in AMOS v.22. Data were missing completely at random (MCAR): Little’s MCAR test (*p* = 0.68).

### Hypothesis testing

In this sample, we did not find evidence that paternal T predicted a father’s self-reported relationship satisfaction (*β* = 0.04, *p* = 0.58).

We moved on to test a model whereby paternal CORT predicted a father’s self-reported relationship satisfaction (covarying for age and time of morning sample). In this CORT model, we also did not find evidence of a relationship between CORT and self-reported relationship satisfaction (*β* = −0.05, *p* = 0.53).

#### DHH

To explore the relationship between the DHH and relationship satisfaction, we created a T × CORT interaction term (i.e., multiplied standardized values of each) and tested a model where this new variable predicted self-reported relationship satisfaction while covarying for the following variables: T, CORT, age, and time of day.

**Figure 1:**
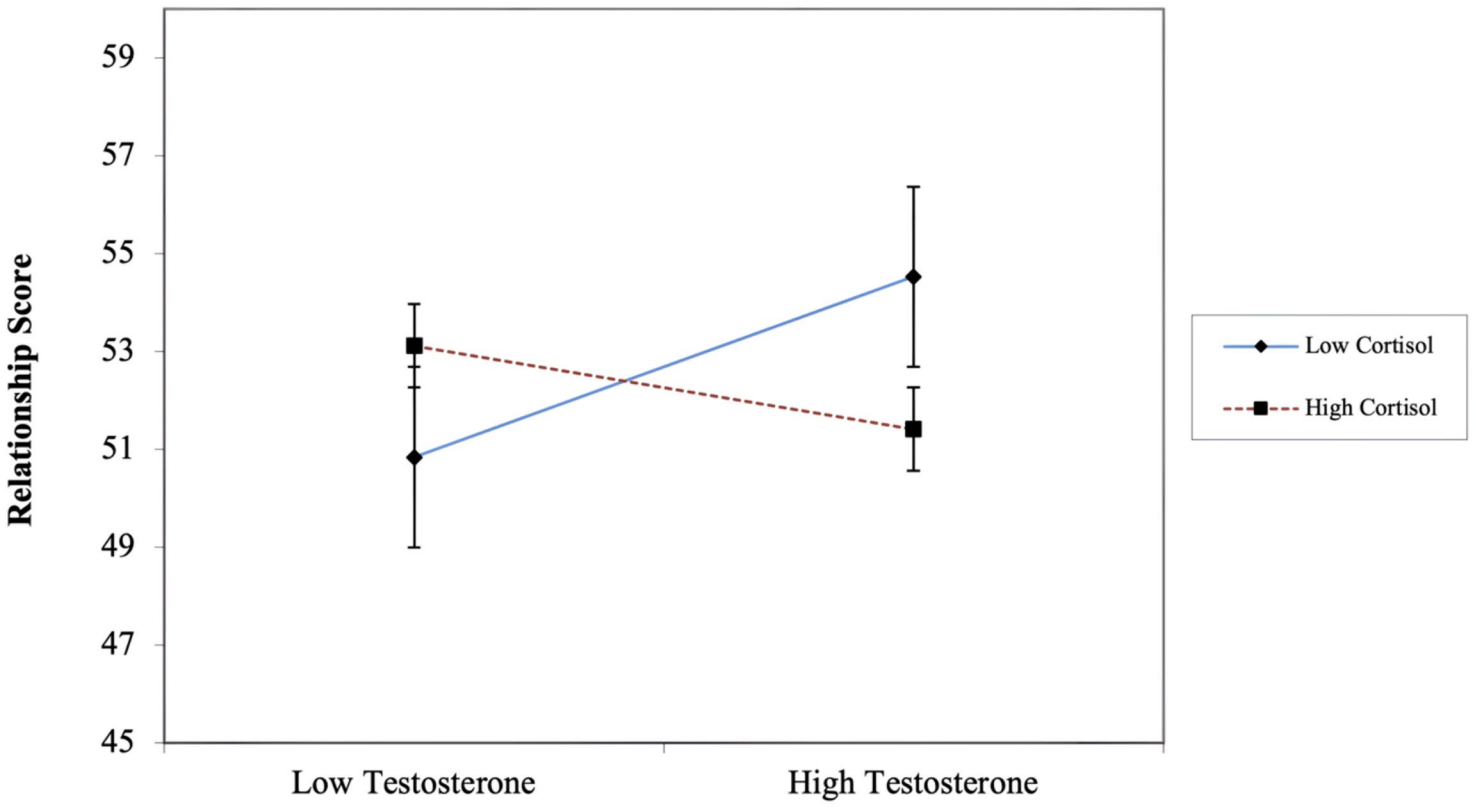
DHH interaction: relationship satisfaction as a function of the interactive effects of T and CORT. Low = 1 SD below mean; high = 1 SD above mean. Intercept and slopes were used to plot relationship satisfaction scores one SD above and below means for T and CORT. Error bars = ± 1 SEM.

We found a small, negative effect for dual hormone influence on relationship satisfaction (*β* = −0.13, *p* = 0.06) (See Table 1 for regression estimates). We created a simple-slope graph^5^ (Figure 1) to aid in interpretation of this small (non-significant) effect. Males with high T and low CORT were more satisfied in their relationships compared to males with lower T^6^.

**Table 1:** Regression model of testosterone T x CORT interaction predicting self-reported relationship satisfaction (*n* = 220 fathers).

	*B*	*β*	SE	CR	*p*
Relationship satisfaction					
T	0.01	0.04	0.02	0.55	0.582
CORT	−2.49	−0.05	3.93	−0.63	0.527
T x CORT	−1.15	−0.13	0.62	−1.87	0.062

B indicates unstandardized regression coefficient. β indicates standardized regression coefficients. SE-standard error. CR-critical ratio.

## Discussion

We expected to find that first-time fathers with high T and low CORT would report lower relationship satisfaction. Instead, our results suggest that this group of fathers have higher relationship satisfaction. Although these findings provide evidence for the DHH being applicable to studies of relationship satisfaction, they are exactly opposite of our prediction. While we speculate on the nature of this small effect below, we caution readers that this result did not reach conventional levels of statistical significance.

Aside from our findings specific to the DHH, we also did not find that T (main effect) nor CORT (main effect) predicted paternal relationship satisfaction. It is not uncommon to find null relationships between single hormones and a given outcome variable while finding effects for the DHH—one of the strengths of a DHH approach. For example, work on T and CORT modulating risk-taking found no significant relationship between each individual hormone and risk-taking but the dual-hormone interaction was found to be significantly related to risk-taking (Mehta et al., 2015). Similarly, dual-hormone research on psychopathy identified a significant relationship between psychopathy and the dual-hormone interaction variable but not with T nor CORT alone (Roy et al., 2019). This pattern is suggestive of how important research on the interaction between the HPA and HPG axis is to fully understand the connectedness of hormones and behavior in interpersonal relationships.

### Mating effort

The effect we report here, while small, requires some speculation to interpret. Traits conventionally associated with high T—dominance, status-seeking, aggression—have been the focus of DHH research and are the same behaviors historically categorized as traits that collectively index one’s mating effort (e.g., Muller and Pilbeam, 2017; Grebe et al., 2019). In this U.S. sample of (mostly) married males, we speculate that mating effort may go beyond behaviors that increase a male’s competitiveness to acquire access to mates but might also capture a psychology aimed at retaining one’s current mate (Barbaro et al., 2016; Salkicevic et al., 2014). While our original interpretation of the literature’s “high T, increased mating effort” relationship led us to predict that high T/low CORT fathers would be less satisfied with their current partner, it is possible that— in humans, a serially monogamous, biparental species (see Donovan et al., 2023 for review)—higher relative androgenicity (specifically, in the postnatal period) may facilitate increased effort directed at one’s current mate. Higher T males may self-report increased satisfaction as they support, retain, secure, elicit additional opportunities from a mate who recently demonstrated her ability (and willingness) to produce offspring. We reiterate the speculative nature of this idea as current theories cannot easily accommodate this possibility. For example, Roney and Gettler’s (2015) model of T modulation within romantic relationships expects that increased baseline T helps males target partners for committed relationships but subsequent elevations in T within a committed relationship may facilitate additional mate seeking (e.g., van Anders et al., 2007). Past research suggests that male T declines after relationship formation and declines further following the arrival of offspring (Gettler et al., 2013). Future research with improved operationalization of mating effort—particularly acquisition vs. retention—is needed.

### Masculinity

The transition to fatherhood presents men with a new identity as fathers. Across cultures the attainment of fatherhood status entails meeting different expectations (Enderstein and Boonzaier, 2015). Involved fathers that take responsibility for paternity and the duties of paternal care are presented with an alternate kind of masculinity (Dunn and Maharaj, 2023; Enderstein and Boonzaier, 2015; Plantin et al., 2003). It is possible that the perception of fatherhood as an enhanced masculine status may help explain the unexpected finding.

### Sexual satisfaction

Sexual satisfaction may further explain this contradictory finding. Sexual activity has been shown to be correlated with higher T, having bidirectional effects in men (van Anders et al., 2007). Fathers who experience milder T declines in response to childbirth have been shown to maintain higher frequencies of sexual activity postpartum (Gettler et al., 2013). Postpartum sexual expectations differ between mothers and fathers (Santtila et al., 2007). This difference between desired amount and actual frequency of sexual behaviors within the relationship dyad can be distressing, indicating that fathers reporting greater sexual satisfaction (i.e., alignment between sexual desire and frequency) may experience lower stress (indexed by CORT) in their relationships (Ahlborg et al., 2005). Future work should include sexual satisfaction to further understand this contradictory finding.

### Limitations

#### Measurement of relationship satisfaction

Satisfaction is a commonly studied variable in the study of close relationships due to the significant impact that it has on downstream behavior (Dhillon et al., 2020). Relationship satisfaction is often collected in the form of a self-report measure where individuals in romantic relationships respond to a questionnaire regarding how they feel about their relationship. Results from these measures have been used as a therapeutic aid (Callaci et al., 2021), to predict postpartum relationship investment (Saxbe et al., 2017a; Saxbe et al., 2017b) or even to assess dyadic endocrine interactions within couples (Edelstein et al., 2014). In this study, we only collected data specific to the relationship satisfaction subscale from Investment Model Scale (Rusbult et al., 1998). The full scale additionally measures commitment level, quality of alternatives, and investment size. While our interests in the current study were exclusively on satisfaction, a more complete understanding of how T, CORT, and their interaction may influence general relationship functioning is an important further consideration. It is possible that the relationships that we uncovered (or did not find) in this study would be different based on our selection of different relationship functioning measures or if we used measures that capture dimensions of relationship functioning beyond mere satisfaction (e.g., close relationship behavior; see Jaremka and Collins, 2017).

#### Hormone collection

Overall, the precision of our results in this study is notably constrained by the number of collection periods and the timing of the morning sample. The salivary analyses in this study only include one saliva sample per participant on a single day in the postnatal period. Despite reliability in T across consecutive days (Dabbs, 1990), our measure of T would have been more precise if we also had assays across consecutive days. Likewise, the same would apply to CORT; despite demonstrated stability across multiple days (Wang et al., 2014), a single collection day is inadequate to securely propose that our findings might replicate in future studies. In addition, the samples were collected within 30 min of awakening but not at the exact moment of waking up which prevents modeling of the awakening response of T and CORT (Kuzawa et al., 2016). Researchers interested in the neuroendocrine functioning of the family unit are thus highly encouraged to plan to collect significantly more samples at highly precise intervals both within and across days (see Kuzawa et al., 2016).

#### Sample characteristics

Another limitation to this study is that we did not collect data on the length of the couple’s relationship. It is likely that reports of relationship satisfaction during the novel stress of transitioning to parenthood will be related to how long ago the relationship began (Farrelly et al., 2015). Additionally, postpartum anxiety and depression were not considered which would have potential to impact paternal hormones. This sample consisted solely of heterosexual couples, predominantly White, well-educated, and middle- to high-income. Although this limits the generalizability of the results, more recent work suggests that there are correlations between T and relationship satisfaction self-reports in parents during the postpartum period in more diverse samples (Cárdenas et al., 2023). Lastly, the inclusion of maternal data (hormones and/or self-reported relationship satisfaction) in future work should add considerable nuance in our understanding how hormones and relationship functioning are related within a dyad (see Edelstein et al., 2014).

#### Biological meaningfulness and statistical significance

This paper uses secondary data from previously published research that focused on T and fatherhood (Corpuz et al., 2021). While the larger project was not originally designed to test for the DHH, the small effect that ran counter to predictions among this community sample may provide valuable insight for scientists interested in applying the DHH to romantic relationships—unexplored terrain in literature that has largely focused on male dominance and risk-taking (Dekkers et al., 2019). A more pressing concern than conventional statistical significance is whether the observed effect size is biologically meaningful. Future experimental work (i.e., exogenous T administration) may reveal thresholds for when CORT meaningfully modulates T in the domain of romantic relationships in a manner predicted by the DHH or other extant neuroendocrine theories. In reporting on and speculating about the direction of the small effect size in this correlational design, we aim to promote further study (experimental, cross-sectional, longitudinal) on the role of hormones in human relationship functioning.

## Conclusion

The novel findings of this report require replication. To our knowledge this is the first study to apply the DHH to relationship satisfaction. Future research would greatly benefit from conducting this study on a longitudinal timeline to increase the number of collection periods for more in-depth analyses of romantic relationship dynamics for parents in the postpartum period. It is important to understand how interactions within the endocrine system are contributing to the postpartum experience. The role of endocrine interactions for T and CORT between as well as within individuals is an important future direction for this work. Future studies have the potential to uncover what makes some romantic relationships more resilient to the challenges of the transition to parenthood than others.

## Data Availability

The data analyzed in this study is subject to the following licenses/restrictions: the data that support the findings of this study are available from the corresponding author RC, upon reasonable request. Requests to access these datasets should be directed to randy.corpuz@pepperdine.edu.

## Footnotes

^1^78.4% of all samples retrieved were collected within 7 days of participant expectorating saliva. There were no differences in assay values from samples picked up within 7 days compared to samples retrieved after 8 + days (all *ps*>0.41).

^2^The average covariance divided by the average variance across all 10 items of the scale.

^3^Salivary endocrine data collection is vulnerable to contextual influences (Granger et al., 2012).

^4^Most other medications listed by fathers in this sample were for allergies (e.g., OTC nasal spray, decongestants), pain (e.g., ibuprofen), or digestive issues (e.g., acid reflux).

^5^As per Dekkers et al. (2019), a negative TxCORT interaction effect is conventionally evidence for dual hormone effects on the outcome variable and should be probed further for interpretation.

^6^Johnson-Neyman intervals were explored using the PROCESS (v4.2) macro (Hayes, 2022) on SPSS (V.30). This technique estimates the values of the moderator (CORT) for which the slope of the predictor (T) on the outcome (relationship satisfaction) will be statistically significant. The corresponding Johnson-Neyman procedure estimated that at values of CORT below 0.48, relationship satisfaction was associated with higher T. No significant associations were observed for CORT levels above 0.48.
